# Acute pancreatitis during pregnancy: maternal and fetal outcomes in a five-case series

**DOI:** 10.11604/pamj.2026.53.122.49731

**Published:** 2026-03-11

**Authors:** Jawaher Hammadi, Hammami Sabra, Ines Mkhinini, Aymen Khalfaoui, Rouis Nour, Ben Jaballah Soukeina, Boukadida Rania, Ouhibi Chayma, Fatnassi Mohamed Ridha

**Affiliations:** 1 Department of Obstetrics and Gynecology, Ibn Aljazzar University Hospital, Kairouan, Tunisia

**Keywords:** Acute pancreatitis, pregnancy, hypertriglyceridemia, biliary lithiasis, maternal outcomes

## Abstract

Pregnancy-related acute pancreatitis (AP) is an uncommon but potentially life-threatening condition for both the mother and the fetus. The most frequent causes are biliary lithiasis and hypertriglyceridemia, while idiopathic and other causes are less common. This case series reports five cases of AP during pregnancy, highlighting the clinical presentation, etiology, management, and outcomes. The series included biliary AP (n=2), hypertriglyceridemia-induced AP (n=1), and idiopathic AP (n=2, including one with persistent symptoms). Maternal age ranged from 24 to 34 years, and gestational age at presentation varied between 10 and 36 weeks. Management involved supportive care, dietary modifications, insulin-heparin therapy, and postpartum cholecystectomy when indicated. All mothers survived, although one case was complicated by recurrent fetal losses due to severe hypertriglyceridemia. Early diagnosis and individualized management of AP during pregnancy are essential to optimizing maternal and fetal outcomes, and this is best achieved through awareness of risk factors, understanding of pregnancy-related physiological changes, and a multidisciplinary approach.

## Introduction

Acute pancreatitis (AP) during pregnancy is rare, occurring in 1 in 1,000 to 1 in 12,000 pregnancies [1]. Maternal susceptibility is increased by hormonal and physiological changes, especially during the third trimester [2]. Estrogen and progesterone promote gallstone formation and can also lead to biliary stasis and hypercholesterolemia. According to Khan *et al*. [3], the second most common cause is hypertriglyceridemia, which is exacerbated by hormonal changes in lipid metabolism. The diagnosis is complicated by the fact that symptoms frequently overlap with obstetric conditions [4]. Five AP cases are presented in this study, emphasizing clinical characteristics, treatment, and maternal-fetal outcomes.

## Methods

**Study design:** this is a retrospective case series of consecutive pregnant patients diagnosed with acute pancreatitis (AP) and managed at the Department of Obstetrics and Gynecology, Ibn Aljazzar University Hospital, Kairouan, Tunisia, between January 2017 and December 2023.

**Setting:** the study was conducted at a tertiary referral obstetrics unit (Ibn Aljazzar University Hospital, Kairouan). Data were collected from hospital medical records, imaging archives, and the obstetrics database.

**Participants (eligibility criteria and selection):** we included all pregnant women admitted to our department during the study period with a diagnosis of acute pancreatitis according to the revised Atlanta criteria (at least two of: 1) typical abdominal pain; 2) serum amylase and/or lipase ≥3x upper reference limit; 3) imaging findings compatible with AP). We excluded non-pregnant patients and patients with incomplete medical records that did not permit confirmation of diagnosis or outcome.

**Variables:** the following variables were extracted: maternal age, parity, gestational age at presentation, presenting symptoms, vital signs, laboratory results (amylase, lipase, C-reactive protein (CRP), white blood cell (WBC), liver tests, triglycerides), imaging performed (ultrasound, computed tomography (CT), magnetic resonance imaging (MRI)), AP etiology (biliary lithiasis, hypertriglyceridemia, idiopathic, other), severity (when available by Balthazar CT score), treatment (supportive, intensive care unit (ICU), insulin-heparin, parenteral nutrition, surgery), maternal outcome (recovery, necrosis, shock, death), fetal outcome (healthy neonate, prematurity, intrauterine fetal death), recurrences and length of follow-up.

**Data sources/measurement:** data were obtained from patient charts and hospital electronic records. Laboratory assays were performed in the hospital laboratory using routine methods. Ultrasonography and CT images were interpreted by the on-call radiologist; when available, the Balthazar grade was recorded from the CT report. For triglycerides, we recorded the peak value during the acute episode.

**Bias:** being a retrospective, single-center series, the study is susceptible to selection bias, information bias (incomplete records), and referral bias (the tertiary center tends to receive more severe cases). We mitigated this by including consecutive cases over the full study period and by cross-checking clinical notes, laboratory records, and imaging reports.

**Study size:** all consecutive eligible cases identified during the 2017-2023 period were included: n = 5.

**Quantitative variables:** continuous variables are reported as ranges and medians where appropriate (e.g., maternal age range 24-34 years). Categorical variables are reported as counts and percentages (e.g., biliary etiology 2/5, 40%). If data were missing for a variable, we report the number missing.

**Statistical methods:** because of the small sample size, analyses are descriptive. We report counts and percentages for categorical variables and ranges (and median if appropriate) for continuous variables. No inferential statistics or multivariable analyses were performed.

**Ethical consideration statement:** data were anonymized prior to analysis. Written informed consent for anonymized data use and for procedures was obtained from the patients when possible. The study protocol was conducted in accordance with the Declaration of Helsinki and was approved by the local institutional ethics committee of Ibn Aljazzar University Hospital.

## Results

Five cases of acute pancreatitis during pregnancy were observed over a five-year period. The patients´ ages ranged from 24 to 34 years, and diagnoses were made between 10 and 36 weeks of gestation. The etiologies included biliary lithiasis, hypertriglyceridemia, and idiopathic causes, with variable clinical severity and generally favorable maternal outcomes of the five patients are summarized in Table 1.

**Table 1:** clinical and outcome characteristics of pregnant women with acute pancreatitis managed at the Department of Obstetrics and Gynecology, Ibn Aljazzar University Hospital, Kairouan (Tunisia), January 2017-December 2023 (N = 5)

Case	Etiology	Trimester	Severity	Treatment	Maternal outcome	Fetal outcome	Follow-up
1	Biliary	Third	Mild	Supportive, cholecystectomy	Recovery	Healthy neonate	18 months, no recurrence
2	Biliary	Third	Mild	Supportive, cholecystectomy	Recovery	Healthy neonate	No recurrence
3	Idiopathic	Third	Severe, necrotizing	ICU, parenteral nutrition	Recovery	Premature but viable	No recurrence
4	Hypertriglyceridemia	Second	Severe	Medical (fasting, insulin-heparin)	Recovery	IUFD, recurrent	Long-term follow-up
5	Persistent idiopathic	Second	Moderate	Supportive	Partial improvement	Healthy neonate	2 years, no recurrence

ICU: intensive care unit; IUFD: intrauterine fetal death

**Participants:** between January 2017 and December 2023, five pregnant women met the inclusion criteria and were included in the analysis (n = 5). There were no additional eligible patients excluded for missing primary diagnosis data. A flow statement (text or simple diagram) may be added showing the number of records screened and included.

**Descriptive data:** patient ages ranged from 24 to 34 years. Gestational age at presentation varied from 10 to 36 weeks. Parity: 2 patients were primigravida and 3 multiparous (report numbers if needed). Comorbidities included a history of prior cesarean(s) or metabolic disease, where applicable (specify any diabetes if present). Presenting symptoms were predominantly severe epigastric pain and vomiting in all cases. Laboratory findings: peak lipase values ranged (report the exact numbers from your file, e.g., case 1 lipase 1500 U/L, case 2 lipase 1800 U/L, etc.). Imaging: abdominal ultrasound was performed in all cases; CT was performed in cases 3 and 4 because of severity/clinical indication, and showed necrotizing pancreatitis in both (Balthazar E). Triglyceride peak values were reported in case 4 as 16-25 g/L (specify exact numbers as in patient notes).

**Outcome data:** maternal outcomes: two cases had necrotizing pancreatitis (cases 3 and 4); one patient experienced maternal shock (case 4). There were no maternal deaths. Management included supportive therapy in all cases, ICU admission and parenteral nutrition for severe necrotizing pancreatitis (cases 3 and 4), and insulin-heparin therapy and dietary measures for hypertriglyceridemia (case 4). Postpartum cholecystectomy was performed in the two biliary cases (cases 1 and 2). Follow-up ranged from 18 months to 2 years with no recurrence in biliary cases.

**Fetal outcomes:** one case had recurrent intrauterine fetal death (case 4; multiple intrauterine fetal demises (IUFDs) in recurrent severe hypertriglyceridemia), one case resulted in a preterm but viable neonate (case 3), and three cases resulted in healthy neonates (cases 1, 2, and 5).

**Main results:** etiology distribution: biliary lithiasis 2/5 (40%), hypertriglyceridemia 1/5 (20%), idiopathic 2/5 (40%). Severity: mild AP in 2/5, severe necrotizing AP in 2/5, and recurrent severe AP with ICU admission in 1/5. Treatments: supportive therapy for all patients; ICU and parenteral nutrition for severe cases; insulin-heparin for hypertriglyceridemia; postpartum laparoscopic cholecystectomy for biliary cases.

**Other analyses:** recurrent episodes were observed in case 4 with progression from biliary to hypertriglyceridemia-related episodes; this patient experienced repeated fetal losses. Long-term follow-up data (up to 2 years) showed no recurrences in the patients who underwent definitive biliary surgery.

## Discussion

Due to symptoms that overlap with those of common obstetric conditions like HELLP syndrome, preeclampsia, and biliary colic, acute pancreatitis during pregnancy can be difficult to diagnose and frequently goes unnoticed [4]. This intricacy is demonstrated by our case series: case 4 necessitated several hospital stays and postponed etiological investigations, whereas case 5 presented with ongoing symptoms from the first trimester that had no apparent cause [1]. Pregnancy-related anatomical changes cause the relocation of abdominal organs, which modifies the location of pain. Amylase and lipase enzyme assays are essential; lipase is more specific, but they cannot identify the cause [5]. Imaging is crucial. Ultrasound is the first option because it is safe and accessible, but its use may be restricted by the size of the uterus or hidden calculi. Despite the risk of fetal radiation, CT is only used in extreme cases (cases 3 and 4). Necrotizing pancreatitis (Balthazar E) was confirmed by CT in case 4, directing treatment while reducing fetal exposure [6]. For a thorough evaluation, MRI is a radiation-free substitute. Once a diagnosis is suspected, identifying the etiology is critical for guiding therapy.

The most frequent cause (cases 1 and 2), accounting for 65-100% of cases, is biliary lithiasis [1,7]. The three main challenges of severe hypertriglyceridemia (case 4) are severe fetal complications, high recurrence risk, and initial lack of recognition [3,8,9].

Dietary restriction, insulin therapy, and lipid monitoring are crucial. Idiopathic cases (cases 3 and 5) draw attention to diagnostic limitations and frequently necessitate postpartum vigilance and extended follow-up [10]. When traditional causes are ruled out, rare causes like autoimmune or drug-induced pancreatitis should be taken into account, particularly in cases of recurrent or unusual presentations [6,11].

Improved maternal-fetal prognosis and customized treatment are made possible by early detection of rare forms [10]. Providing multidisciplinary care is crucial. Supportive therapy (fluid resuscitation, analgesia, nutrition) is universal, even though etiology-specific interventions include postpartum cholecystectomy for biliary AP, insulin-heparin therapy for hypertriglyceridemia, and close monitoring for persistent idiopathic cases. Plasmapheresis or intensive care unit treatment might be required in extreme circumstances [11]. Patients with hypertriglyceridemia require dietary counseling, lifestyle modifications, and long-term lipid monitoring to prevent recurrence, especially in subsequent pregnancies. Contraception and family planning are crucial for reducing future risks. Maternal mortality is now less than 3%. Prematurity, intrauterine growth restriction, and IUFD are among the risks for the fetus, especially in cases of severe hypertriglyceridemia. Long-term monitoring is required for persistent symptoms (case 5) in order to protect the health of both the mother and the fetus. In conclusion, high clinical suspicion, prompt diagnostic assessment, and specialized treatment are necessary for AP during pregnancy. Optimizing maternal and fetal outcomes requires etiology-specific therapy, careful imaging use, and awareness of physiological changes [11].

The main limitation of this work is the small number of cases and its retrospective, single-center nature, which may limit the generalizability of the results. Additionally, long-term maternal metabolic outcomes were not systematically assessed.

**Limitations:**this study has several limitations: it is retrospective and single-center, with a small sample size (n = 5), which limits generalizability and prevents inferential statistical analysis. The retrospective design may have led to incomplete data capture and information bias. Referral bias is also possible since a tertiary center is more likely to admit severe cases. Finally, long-term maternal metabolic outcomes (lipid control after discharge) were not systematically assessed.

## Conclusion

Despite being uncommon, acute pancreatitis during pregnancy poses serious risks to both the mother and the fetus. Therapy tailored to the etiology, supportive care, and early detection is crucial. Results are best achieved with multidisciplinary care and close postpartum monitoring, especially in cases of severe hypertriglyceridemia or chronic idiopathic conditions. Awareness of risk factors and timely intervention are essential to minimize maternal and fetal complications

### What is known about this topic

- Acute pancreatitis during pregnancy is rare but can be life-threatening for both mother and fetus;
- Biliary lithiasis and hypertriglyceridemia are the most common causes;
- Severe cases may lead to maternal complications, preterm delivery, or fetal loss.

### What this study adds

- This study highlights the clinical presentation, management, and outcomes of five cases, including idiopathic and recurrent forms;
- It emphasizes the importance of early diagnosis, multidisciplinary care, and etiology-specific treatment;
- And it provides long-term follow-up data, showing generally favorable maternal outcomes and variable fetal outcomes.
